# Epigenetic switch reveals CRISPR/Cas9 response to cytosine methylation in plants

**DOI:** 10.1111/nph.18405

**Published:** 2022-08-18

**Authors:** Sarah Raffan, Navneet Kaur, Nigel G. Halford

**Affiliations:** ^1^ Rothamsted Research Harpenden AL5 2JQ UK

**Keywords:** CRISPR/Cas9, crop improvement, cytosine methylation, DNA repair, epigenetic switch, gene regulation and genome stability, heterochromatin and euchromatin, mutation frequency

## Abstract

This article is a Commentary on Přibylová *et al*. (2022) **235**: 2285–2299.

Genome editing techniques, such as the CRISPR/Cas9 system, offer a game‐changing opportunity for crop improvement by enabling precise modifications to be made at targeted genomic loci. The CRISPR/Cas9 system has been employed successfully in many plant species; however, in order to use the system to its full potential, it is important to understand precisely how it functions and the factors that may limit its effectiveness. The mechanistic details of Cas9‐induced double‐strand breaks (DSBs), that underpin the mutational ability of the system, have been well‐described. It also is known that the efficiency of editing varies for different target sequences. However, the impact of epigenetic modifications on CRISPR/Cas9 efficacy and subsequent DNA repair is poorly understood, especially in plants.

‘Cytosine methylation was not the only factor that affected CRISPR/Cas9 efficiency in the study, with virus infection itself increasing the mutation rate.’

Epigenetic modifications affect gene regulation and genome stability. As such, the epigenetic status of an editing target site may influence the frequency of CRISPR/Cas9‐induced mutations, for example, by affecting how well Cas9 binds and cuts, or the efficiency and accuracy of DNA repair mechanisms. Genome‐wide analyses have shown the efficiency of CRISPR/Cas9 editing to be lower for heterochromatin than euchromatin (Daer *et al*., 2017), although this is still contested (Kallimasioti‐Pazi *et al*., 2018). However, comparisons of editing efficiencies between heterochromatin and euchromatin have involved the analysis of two or more loci, and different target loci vary in more than just their epigenetic status. In this issue of *New Phytologist*, Přibylová *et al*. (2022; pp. 2285–2299) investigated the effect of cytosine methylation on the generation of CRISPR/Cas9‐induced mutations at multiple target sites within the same locus in *Nicotiana benthamiana,* using a virus‐based epigenetic switch. This epigenetic switch allows for the conversion of the chromatin state of the target locus and so can be used to shed light on how cytosine methylation affects the frequency and outcome of CRISPR/Cas9‐induced editing at a single site. The authors also highlighted the important role of single‐nucleotide microhomology‐mediated DNA repair in genome editing.

## gRNA: on‐target capability

Successful editing relies on the selection of efficient gRNAs, and on how the nucleotide sequence of a target site influences the editing outcome, with some targets being more prone to mutagenesis than others. This may be a consequence of differences in Cas9 recognition, DSB formation or the error‐rate of DNA repair. Interestingly, the study showed that targeting the same site but on complementary DNA strands produced different mutagenesis rates, highlighting the intrinsic effect of the underlying sequence. There are many bioinformatic tools available to help researchers identify ‘good’ target sequences in order to achieve better editing efficiency, while minimizing the potential for off‐target effects. However, the predicted on‐target efficiencies for these tools can often be inaccurate (Naim *et al*., 2020), possibly, at least in part, because these tools only consider genetic influences and not epigenetic factors.

## Epigenetic switch: changing the chromatin status at a targeted site

**Fig. 1:**
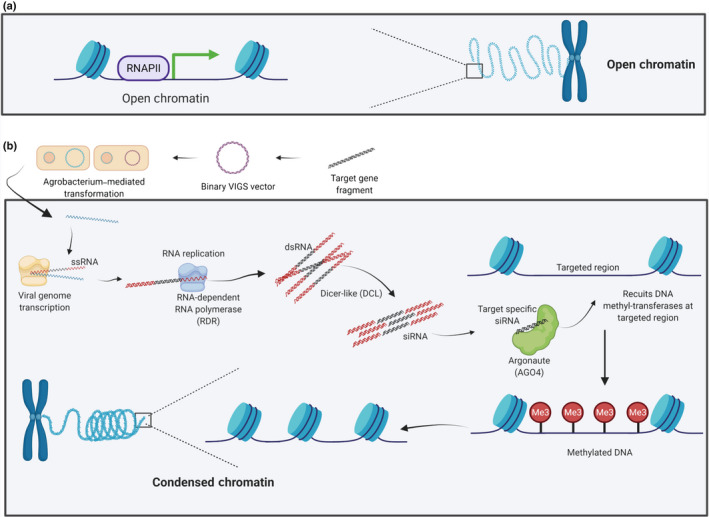
Schematic overview of the molecular mechanism of the RNA‐dependent DNA methylation (RdDM) pathway. (a) Open chromatin representing active transcription. (b) In order to induce the RdDM pathway, a recombinant viral DNA is developed by adding a fragment of target region into a *Tobacco rattle virus* (TRV) VIGS vector and transforming the vector into *Agrobacterium tumefaciens*. Upon infection, T‐DNA containing the recombinant viral genome is transcribed by the host’s RNA polymerase (in yellow) to produce single stranded RNA (ssRNA), which then is converted to double‐stranded RNA (dsRNA) by RNA‐dependent RNA polymerase (in grey). DICER‐like enzymes recognize the dsRNA molecules and cleave them into short, interfering RNAs (siRNA). Argonaute (AGO4; in green) with target‐specific virus‐derived, siRNAs, directs plant‐encoded DNA methyltransferases to methylate cytosine residues in DNA at sites with sequence complementarity to the siRNA. Finally, DNA methylation induces chromatin condensation. [Colour figure can be viewed at wileyonlinelibrary.com]

Přibylová and colleagues in this study used a recombinant *Tobacco rattle virus* (TRV) virus‐induced gene silencing (VIGS) vector (Jones *et al*., 2001) to trigger the plant’s RNA‐dependent DNA methylation (RdDM) pathway (Fig. 1), inducing DNA methylation at the target locus. Thus, they were able to investigate the direct effect of DNA cytosine methylation on CRISPR/Cas9 by comparing the rate of induced mutations at a single site, with or without methylation. The VIGS vector targeted the 35S promoter and green fluorescent protein (GFP) coding region (Fei *et al*., 2021) in a well‐characterized transgenic *N. benthamiana* line (named 16c) (Ruiz *et al*., 1998). The use of this transgenic line allowed for a visual assessment of how effective the epigenetic switch was at inducing DNA methylation and transcriptional silencing, which was then confirmed by quantitative reverse transcription (qRT)qPCR and bisulfite sequencing. The progeny of the infected plants were also investigated because they carried the same induced methylation patterns but were not infected with the virus (Jones *et al*., 2001).

## How does DNA methylation affect CRISPR/Cas9 efficacy?

Cytosine methylation affected the ability of the CRISPR/Cas9 system to induce mutations in a target‐site specific manner. The higher the level of cytosine methylation for targeted regions in the promoter region, the lower the levels of CRISPR/Cas9 editing. By contrast, despite heavy methylation, there was no significant change in mutagenesis frequency in the coding region. Přibylová and colleagues suggest that DNA changes associated with promoter methylation might make the DNA less accessible for the CRISPR/Cas9 machinery: something that has also been observed in mammalian cells (Schep *et al*., 2021). In coding regions, however, ongoing chromatin remodelling driven by active gene transcription (Farnung *et al*., 2021) might prevent methylation‐induced changes in the chromatin structure, allowing increased access of the CRISPR/Cas9 machinery to the DNA. Transcription itself might also affect editing efficiency, potentially by interfering with the CRISPR/Cas9 machinery, which would explain the higher levels of mutations observed in transcriptionally silenced plants compared with transcriptionally active plants. This has also been observed in human cells (Daer *et al*., 2017).

Cytosine methylation was not the only factor that affected CRISPR/Cas9 efficiency in the study, with virus infection itself increasing the mutation rate. Stress may induce a higher frequency of mutations by reducing the effectiveness of the plant’s DNA repair mechanisms. The level of expression of CRISPR/Cas9 components or the number of cells successfully transformed also affected mutational frequency, with increased *Agrobacterium* density leading to increased mutagenesis.

DNA methylation also affected the mutational outcome of editing, with RdDM changing the relative frequency of insertions and deletions. Cas9 nuclease is known to introduce both blunt‐ and staggered‐ended DSBs, with the latter resulting in single nucleotide insertions (Jinek *et al*., 2012; Lemos *et al*., 2018). The study illustrated that the frequency of staggered DSBs is affected by both genetic and epigenetic factors. Přibylová and colleagues propose that physical tension of the DNA may affect whether a blunt‐ or staggered‐end cut is produced, and DNA tension may be influenced by the local chromatin state. Small nucleotide insertions of the type observed here have also been seen in other plant species (Nekrasov *et al*., 2013; Li *et al*., 2018; Lee *et al*., 2019; Raffan *et al*., 2021).

The epigenetic status of the target site also influenced the type of deletions that were produced, with the majority of deletions occurring in the protospacer adjacent motif (PAM) distal region. This region is released first from the CRISPR/Cas9 complex and may be more exposed to exonucleases. Protospacer adjacent motif‐distal cleavages can be repaired by 5′ DNA single‐nucleotide microhomology‐mediated DNA repair, which is distinct from the MMEJ Pathway, and this could be an important factor to consider when designing potential gRNAs.

Přibylová and colleagues concluded that DNA methylation impacts upon CRISPR/Cas9 mutation frequency and outcome. Furthermore, changes in the relative frequency of insertions and deletions, and the involvement of 5′ DNA single nucleotide‐mediated DNA repair could potentially be exploited with better target selection and gRNA design, with possible future applications in plants and beyond. Further investigations will be required to elucidate the underpinning molecular mechanisms driving this methylation effect and elucidating the DNA repair pathways that are involved, and it will be fascinating to see how this research unfolds.
